# A Rare Cause of Intellectual Disability

**DOI:** 10.7759/cureus.51451

**Published:** 2024-01-01

**Authors:** Íris Oliveira, Andreia Fernandes, Mafalda Pereira, Márcia Rodrigues, Noémia Silva, Carla Mendonça

**Affiliations:** 1 Pediatrics, Centro Hospitalar Universitário do Algarve - Unidade de Faro, Faro, PRT; 2 Service of Medical Genetics, Department of Pediatrics, Centro Hospitalar Universitário Lisboa Norte - Hospital Santa Maria, Lisboa, PRT

**Keywords:** language impairment, microcephaly, delayed psychomotor development, dyrk1a, mrd7

## Abstract

A seven-year-old female was followed in a developmental clinic from the age of nine months due to delayed psychomotor development. The first physical examination showed a newborn with irritability and a large anterior fontanelle. A transfontanellar ultrasound was performed, revealing mild enlargement of the lateral and third ventricles. Head circumference remained below the third percentile until the age of five months, then rose to the third percentile. Developmental milestones were globally delayed, with expressive language being more severely affected and axial hypotonia with appendicular hypertonia on neurological examination. Subsequent medical observation revealed deep-set eyes, mildly up-slanted palpebral fissures, a high nasal bridge with a broad nasal tip, a thin upper lip, widely spaced teeth, retrognathia, and a slight pectus excavatum. Genetic investigation revealed the diagnosis, with whole-exome sequencing consistent with the genetic diagnosis of autosomal dominant mental retardation type 7 (MRD7).

All patients diagnosed with MRD7 have a development delay detected at a young age and, typically, a mild to severe intellectual disability later in life. All individuals present language impairment, especially in verbal expression. Motor development is typically affected by gait disturbances and generalized hypertonia, which are noted early in life. Microcephaly is a prominent feature of this syndrome, present in over 90% of the cases. The most common findings in MRD7 (microcephaly and intellectual disability) have a broad differential diagnosis. Some disorders have multiple findings in common with MRD7, such as Angelman syndrome (AS), MECP2 disorders, or Mowat-Wilson syndrome (MWS).

MRD7 is a rare genetic syndrome characterized by developmental delay/intellectual disability, microcephaly, autism spectrum disorder, behavior problems, typical facial features, and seizures. Early intervention is more likely to be effective and potentially change a child’s developmental path. Small gains early in life could represent a significant difference in the children’s future autonomy.

## Introduction

Autosomal dominant mental retardation type 7 (MRD7) incidence is less than 1/1,000,000 and accounts for 0.1-0.5% of individuals with intellectual disability and/or autism [1]. All patients diagnosed with MRD7 have a development delay detected at a young age and, typically, a mild to severe intellectual disability later in life. All individuals present language impairment, especially in verbal expression. Only some individuals learn how to speak. Motor development is typically affected by gait disturbances and generalized hypertonia, which are noted early in life. Independent walking is usually achieved after two to three years of age. Feeding difficulties occur in the vast majority and may continue through adulthood [2].

Microcephaly is a prominent feature of this syndrome, present in over 90% of the cases. Other growth features include intrauterine growth restriction and/or oligohydramnios, low weight, or short stature [2]. Signs that indicate global cerebral underdevelopment/hypomyelination can be detected by brain imaging, for example, cortical brain atrophy, enlarged ventricles, or hypoplasia of the corpus callosum. About 50% of patients develop epilepsy (some only have febrile seizures during infancy) [2].

Many children have autism spectrum disorder and other behavioral problems, such as night awakening, trouble falling asleep, hyperactivity, stereotypies, or anxiety [3]. Affected individuals have a distinctive facial gestalt of deep-set eyes, mild upslanting palpebral fissures, a short nose with a broad tip, dysplastic ears, and retrognathia with a broad chin. Later in life, the nose may acquire a more prominent appearance [2]. Other associated features include eye abnormalities (strabismus, refractive error, and optic nerve hypoplasia), urogenital anomalies (hypoplastic/shawl scrotum, undescended testes, micropenis, hypospadias, frequent urinary infections, vesicoureteral reflux, and unilateral renal agenesis), cardiac defects (septal abnormalities, valve defects, patent ductus arteriosus, or hypoplastic left heart), musculoskeletal features (pectus excavatum, scoliosis, kyphosis and hand/foot abnormalities), and dental anomalies (widely spaced teeth, neonatal teeth, delayed primary dentition and hardened dental plaque) [1,2].

## Case presentation

A seven-year-old female was followed in a developmental clinic from the age of nine months due to delayed psychomotor development. She was born after an uncomplicated full-term pregnancy and vaginal delivery. Newborn screening was normal. Family history was non-contributory.

**Figure 1:**
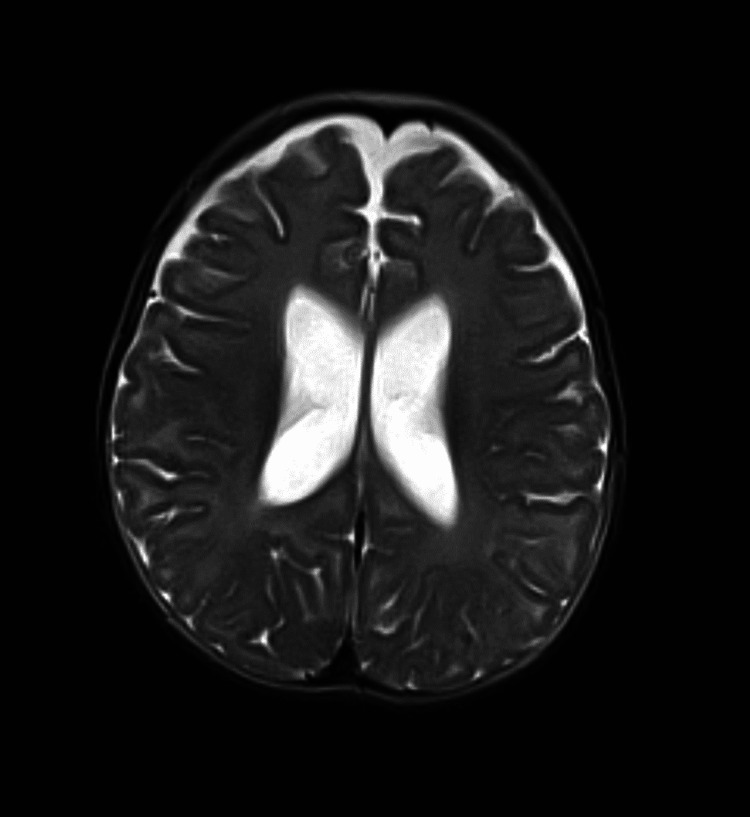
Mild enlargement of lateral ventricles

A physical examination during the first day of life showed a newborn with irritability and a large anterior fontanelle. A transfontanellar ultrasound was performed that same day, revealing mild enlargement of the lateral and third ventricles, later confirmed at five months of age by magnetic resonance imaging (Figure 1).

**Figure 2:**
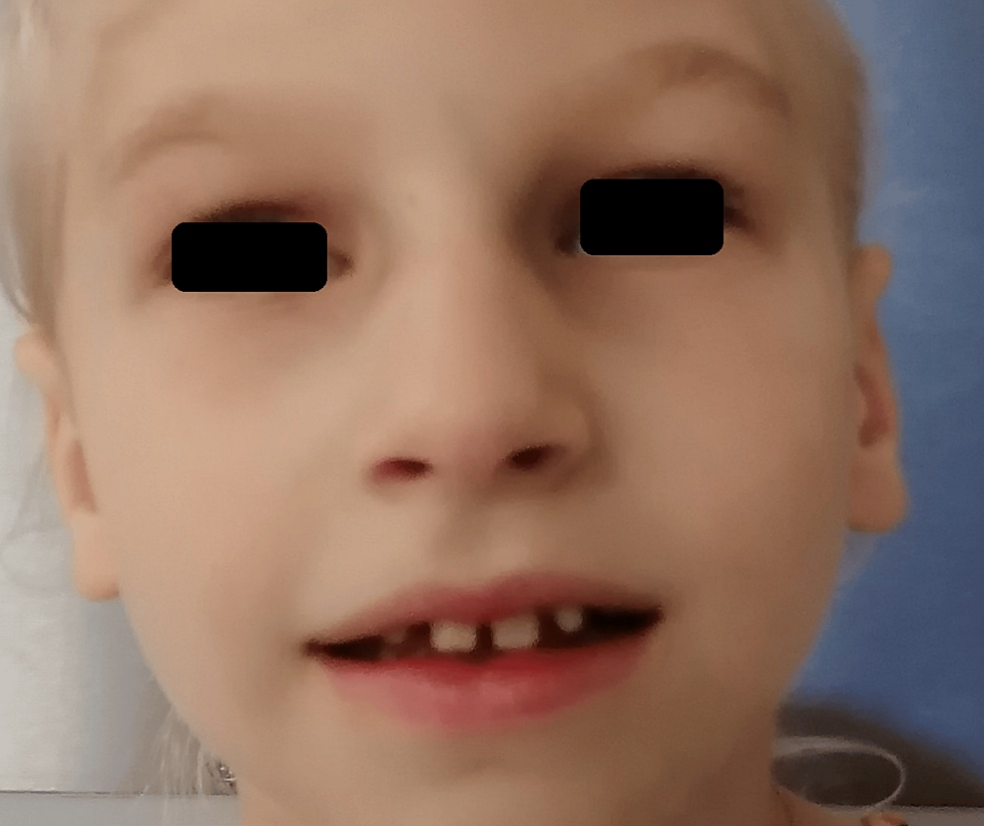
Facial characteristics of a patient diagnosed with MRD7 - front

**Figure 3:**
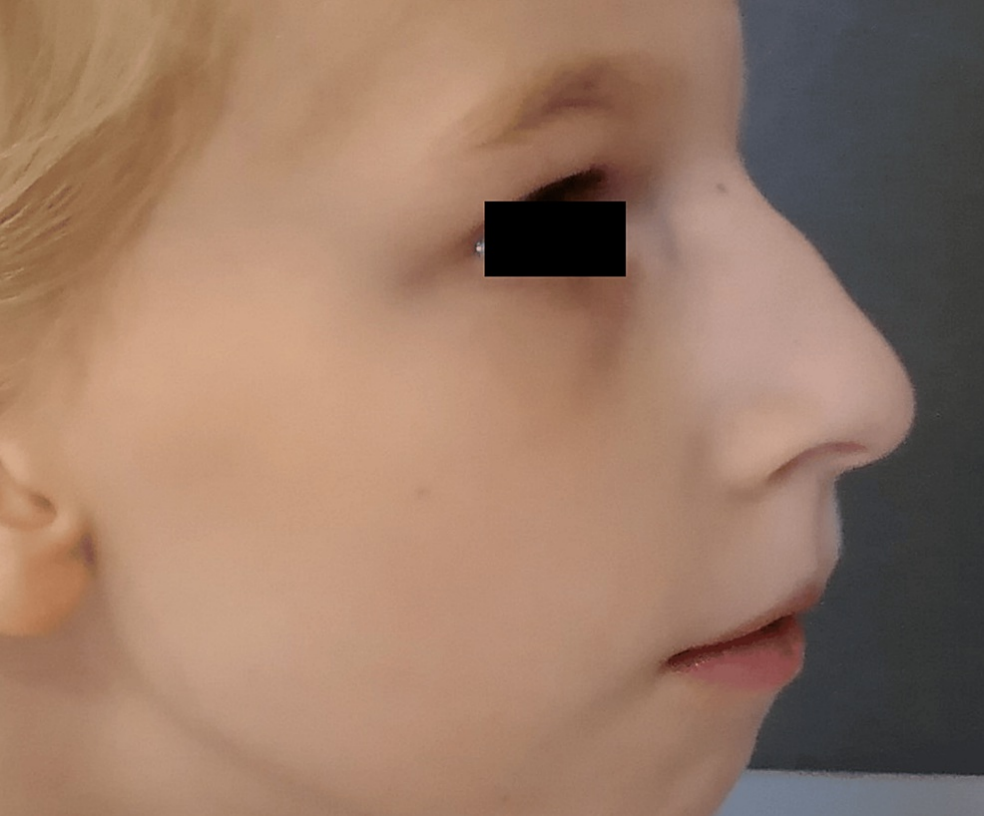
Facial characteristics of a patient diagnosed with MRD7 - profile

Head circumference remained below the third percentile until the age of five months, then rose to the third percentile. During the first years of life, developmental milestones were globally delayed, with expressive language being more severely affected and axial hypotonia and appendicular hypertonia on neurological examination. Concerning her behavior, she had periods of irritability, sleep disturbances, and stereotypies. Swallowing difficulties appeared with the introduction of solids when she was six months old. In the first year of life, she had frequent urinary tract infections with no underlying urogenital malformation. Subsequent medical observation revealed deep-set eyes, mildly upslanted palpebral fissures, a high nasal bridge with a broad nasal tip, a thin upper lip, widely spaced teeth, retrognathia, and a slight pectus excavatum (Figures 2-3).

She was evaluated for the first time at a genetics appointment when she was three years old. A genetic investigation revealed the diagnosis. An array-based comparative genomic hybridization was performed and showed no copy number variants. Further genetic investigation with whole-exome sequencing identified a heterozygous likely pathogenic variant [c.894del, p.(Lys299Asnfs*7)] in the DYRK1A gene, consistent with the genetic diagnosis of autosomal dominant MRD7. The DYRK1A gene encodes the DYRK1A protein, highly expressed in the developing central nervous system, whose haploinsufficiency causes DYRK1A-related intellectual disability syndrome.

## Discussion

The patient has several features that characterize MDR7’s broad syndromic phenotype. As part of the evaluation that led to the diagnosis, she was evaluated concerning her development, feeding problems, urogenital, cardiovascular, ocular, and neurologic abnormalities, and genetic counseling. At the age of nine months, she initiated physical, speech, and occupational therapy, which she maintains until today. As a result of an early intervention, she started walking without support at the age of 20 months. After starting to talk at 17 months of age, she is now capable of expressing herself through three-word sentences. She has become progressively more autonomous regarding her personal hygiene and feeding. Despite showing some difficulty in concentrating, she continues to make progress at school.

There are more than 2000 genes linked to intellectual disability. The most common findings in MRD7 (microcephaly and intellectual disability) have a broad differential diagnosis [1]. Some disorders have multiple findings in common with MRD7, such as Angelman Syndrome (AS), MECP2 disorders, or Mowat-Wilson Syndrome (MWS) [4]. Unlike MRD7, AS is characterized by a specific EEG pattern, unique behavior and facial gestalt. Microcephaly in AS is also less severe than in MRD7. In MECP2 disorders, a developmental regression is observed, contrasting with the MRD7 developmental path. Finally, MWS is more likely to be associated with several malformations [2].

## Conclusions

MRD7 is a rare genetic syndrome characterized by developmental delay/intellectual disability, microcephaly, autism spectrum disorder, behavior problems, typical facial features, and seizures. Diagnosis is established by the identification of a heterozygous pathogenic variant in the DYRK1A gene in an individual with suggestive findings. Greater awareness of MRD7 among healthcare professionals may allow an early diagnosis and, consequently, the multidisciplinary follow-up that patients with this syndrome require. Early intervention is more likely to be effective and potentially change a child’s developmental path. Small gains early in life could represent a significant difference in the children’s future autonomy.
